# Multisystem Inflammatory Syndrome in Children (MIS-C) Associated with 2019 Novel Coronavirus (SARS-CoV-2) Infection

**DOI:** 10.1155/2020/8875987

**Published:** 2020-07-18

**Authors:** Helen Kest, Ashlesha Kaushik, William DeBruin, Mario Colletti, David Goldberg

**Affiliations:** ^1^Pediatric Infectious Disease, Department of Pediatrics, St. Joseph's Children's Hospital, Paterson, NJ 07503, USA; ^2^Pediatric Infectious Diseases, Unity Point Health, University of Iowa, Carver College of Medicine, 2720 Stone Park Blvd, Sioux City, IA 51104, USA; ^3^Pediatric Intensive Care Unit, Department of Pediatrics, St. Joseph's Children's Hospital, Paterson, NJ 07503, USA

## Abstract

We report three critically ill pediatric patients (aged 6–10 years), presenting with features of multisystem inflammatory syndrome in children (MIS-C) from April 4 to May 10, 2020, to a tertiary-care center in New Jersey, United States. All patients tested positive for severe acute respiratory syndrome coronavirus 2 (SARS-CoV-2) infection and were previously healthy. Clinical presentations were similar with fever, abdominal pain, gastrointestinal complaints, and/or rash. One patient had altered mental status with cerebrospinal fluid (CSF) findings consistent with aseptic meningitis. Laboratory values were remarkable for high levels of C-reactive protein, D-dimers, B-type natriuretic peptide (BNP), and troponin in all patients. All had low albumin levels. Evaluation for other infectious etiologies was negative. All of the patients were critically ill, requiring admission to the intensive care unit. All had circulatory shock and needed inotropes. Two patients had respiratory failure requiring advanced respiratory support and one had cardiac dysfunction. All patients received steroids, and two received intravenous immunoglobulin (IVIG). One patient received tocilizumab. None of the children died. MIS-C is a recently recognized pediatric illness spectrum in association with SARS-CoV-2 infection, and clinical characterization is essential for understanding disease mechanisms to inform clinical practice.

## 1. Introduction

We are in the midst of an unprecedented global pandemic of coronavirus disease (COVID-19), caused by the novel coronavirus SARS-CoV-2. Initially thought to affect children less severely [1–3], SARS-CoV-2 infection has recently been associated with a novel set of clinical manifestations presently called multisystem inflammatory syndrome in children (MIS-C) [4] that is beginning to be recognized in the United States, although published reports from the United States are lacking. We describe three critically ill patients with the spectrum of MIS-C associated with SARS-CoV-2 infection presenting to a tertiary-care center in New Jersey.

## 2. Case 1

A 10-year-old female with no significant past medical history presented following four days of febrile illness (fever to 105°F), associated with progressively worsening diffuse abdominal pain and multiple episodes (4-5 times per day) of watery, nonbloody, nonmucoid stools. One day prior to presentation, she developed pink eyes and generalized rash. There was associated lethargy but no report of chest pain, shortness of breath, or vomiting. There were no sick contacts. Upon presentation to the pediatric emergency department (ED), the patient was noted to be lethargic and febrile, with a temperature of 38.8°C and heart rate of 144 beats per minute. Oxygen saturation and blood pressure were normal. Physical examination was remarkable for conjunctival injection, a generalized blanching rash, and diffuse abdominal tenderness. Four hours after admission, she became hypotensive (blood pressure of 82/43 mmHg) needing multiple fluid boluses and inotropic support with norepinephrine. Blood and urine cultures were obtained and were negative. The patient was noted to have neutrophilia, lymphopenia, hypoalbuminemia, elevated ESR (erythrocyte sedimentation rate), CRP (C-reactive protein), fibrinogen, D-dimers, ferritin, troponin, and B-type natriuretic peptide (BNP). Clinical characteristics and laboratory evaluation are summarized in Table 1.

Additionally, the patient had a negative respiratory viral panel (RVP). SARS-CoV-2 RT-PCR (reverse-transcription polymerase chain reaction) was positive. She received intravenous antibiotics (ceftriaxone and linezolid) for a total of 2 days until blood, urine, and stool cultures were negative. Imaging studies were unremarkable (Table 1). She received IVIG (intravenous immunoglobulin at 2 g/Kg), steroids, and enoxaparin for treatment and was discharged home after 6 days of hospital stay.

## 3. Case 2

A 6-year-old female with no significant past medical history presented with fever up to 39°C for four days with associated headaches, vomiting, abdominal pain, diarrhea, conjunctivitis, and rash. She had exposure to grandfather with COVID-19 two weeks earlier. At admission, she had a temperature of 40°C, heart rate of 138 beats per minute, respiratory rate of 24 breaths per minute, and oxygen saturation of 100% on room air. Two hours later, she was noted to be febrile to 40°C and hypotensive with blood pressure of 70/40 mmHg requiring pressor support and developed respiratory failure needing intubation and mechanical ventilation. The patient was noted to have lymphopenia, thrombocytopenia, hypoalbuminemia, elevated levels of inflammatory markers, D-dimers, ferritin, troponin, and BNP as shown in Table 1. The patient had a negative respiratory viral panel (RVP) and SARS-CoV-2 PCR. Antibody testing for SARS-CoV-2 (SARS-CoV-2 IgG) was positive.

The patient received broad spectrum intravenous antibiotics (meropenem and linezolid), IVIG (2 g/kg), methylprednisolone, and enoxaparin. However, due to pulmonary edema and increased oxygen requirements, she was treated with tocilizumab (one dose of 12 mg/kg) on day 4 of hospitalization. She was extubated on day 9 of hospitalization and gradually weaned off oxygen. She was discharged after a hospital stay of 14 days.

## 4. Case 3

A 9-year-old previously healthy male presented with 4 days of febrile illness (to 38.2°C) with alerted mental status, diarrhea, vomiting, conjunctivitis, shortness of breath, and facial swelling. He was admitted to the pediatric intensive care unit (PICU) and intubated for hypoxic respiratory failure. An echocardiogram showed borderline low systolic function with a shortening fraction of 29–30%. He was treated with dopamine and milrinone and required respiratory support with bilevel positive airway pressure (BiPAP). The patient was noted to have leukocytosis, neutrophilia, and hypoalbuminemia, with elevated levels of troponin and BNP (Table 1). The cerebrospinal fluid (CSF) findings were consistent with aseptic meningitis (white cell-count of 100/*μ*L with 52% lymphocytes, 36% monocytes, a normal glucose of 84 mg/dL (normal range 50 to 75 mg/dL), and protein of 31 mg/dL (normal range 15–45 mg/dL). The patient had a negative respiratory viral panel (RVP) and a positive SARS-CoV-2 RT-PCR.

The hospital course was complicated by impairment of kidney function manifested by high serum creatinine which resolved with hydration. The patient was treated with broad-spectrum intravenous antibiotics (ceftriaxone and linezolid) as well as hydroxychloroquine, methylprednisolone, and enoxaparin. He became afebrile on day 3 of hospitalization. Blood, CSF, and urine cultures were negative. Testing for Lyme, *Mycoplasma pneumoniae*, cytomegalovirus (CMV), West Nile virus, and Epstein Barr virus (EBV) were all negative.

## 5. Discussion

This is the first report of multisystem inflammatory syndrome in children (MIS-C) associated with SARS-CoV-2 infection in New Jersey in three hospitalized patients from April 4 to May 10, 2020. All patients (aged 6–10 years) were previously healthy, over 50^th^ centile for weight with no comorbidities, who developed multiorgan involvement and systemic inflammation leading to critical illness needing intensive care.

MIS-C is a newly recognized spectrum of disease manifestations in children associated with novel coronavirus SARS-CoV-2 infection [4]. MIS-C appears to exclusively affect children and has been recently recognized with few published international reports [5–7]. Features of Kawasaki disease- (KD-) like illness were first described in children with 2019 coronavirus disease (COVID-19) in the United Kingdom (UK) in late April and a recent study reported children with cardiac involvement and shock of whom one died [5, 6]. The study from Italy also described KD-like features in children with SARS-CoV-2 infection [7]. The condition is being recently recognized in the United States; however, there is lack of published reports in the US. The Centers for Disease Control and Prevention (CDC) has declared MIS-C to be a reportable illness as of May 14, 2020, and has recently provided a case definition which includes *patients under 21 years of age with fever (>38.0°C for ≥24 hours, or report of subjective fever lasting ≥24 hours), laboratory evidence of inflammation [one or more of the following: elevated C-reactive protein (CRP), erythrocyte sedimentation rate (ESR), fibrinogen, procalcitonin, D-dimer, ferritin, lactic acid dehydrogenase (LDH), or interleukin 6 (IL-6), elevated neutrophils, reduced lymphocytes, and low albumin)], severe illness needing hospitalization, and involvement of two or more organ systems (cardiac, renal, respiratory, hematologic, gastrointestinal, dermatologic, or neurological), with positive testing for SARS-CoV-2 indicating current or recent infection or COVID-19 exposure; and no other alternative plausible diagnoses* [4].

All of our patients presented with multisystem disease with elevated inflammatory markers, consistent with the CDC case definition of MIS-C. Testing to detect SARS-CoV-2 infection was positive in all patients. The report from Italy described positive testing by RT-PCR (reverse-transcription polymerase chain reaction) and/or serology for SARS-CoV-2 similar to our observation [7], although in the study from the UK, all children were antibody positive [5]. Evaluation for other infectious agents was negative.

All of our patients had circulatory shock requiring inotropic support and all had elevated BNP and troponin levels, similar to study from the UK. [5]. However, one of our patients presented with neurologic involvement which has not been observed in published reports thus far.

Majority of the children in reports thus far showed recovery with response to varying degrees of intensive care, with most requiring respiratory support, inotropes, IVIG (intravenous immunoglobulin) and steroids [5–7], as seen in our patients. One of our patients received tocilizumab unlike any of the previous reports of MIS-C. Interleukin-6 inhibitors may be beneficial given the cytokine storm associated with COVID-19; however, their role in treatment of MIS-C needs to be further investigated. None of the children died in this report similar to the study from Italy [7], and all of the children showed recovery and are being followed up for long-term complications.

Clinical presentation of MIS-C with multiorgan involvement and elevated inflammatory markers may have overlapping features with Kawasaki disease and toxic shock syndrome, but is presently understood to be a separate phenomenon. MIS-C is thought to be related to a post-viral immune-mediated inflammatory process, as suggested by recognition of clinical cases as we move into post-peak phase of COVID-19 illness incidence, although the pathogenesis of the syndrome remains largely unclear [4].

## 6. Conclusion

We observed multisystem inflammatory syndrome in children (MIS-C) in three previously healthy patients with SARS-CoV-2 infection, all of whom became critically ill with multisystem involvement. SARS-CoV-2 associated MIS-C requires further investigation. As knowledge about novel manifestations of COVID-19 in children is evolving, reporting is essential to better equip clinicians in recognizing the spectrum of symptoms of MIS-C, which is imperative for timely initiation of appropriate management.

## Figures and Tables

**Table 1:** Clinical characteristics of patients.

	Patient 1	Patient 2	Patient 3
*Patient characteristics*			
Age (years)	10	6	9
Sex	Female	Female	Male
Race	White Hispanic	White Hispanic	African American
*Time to presentation*	4 days	4 days	4 days
*SARS-CoV-2 testing (RT-PCR)*	Positive	Negative RT-PCR; positive SARS-CoV-2 IgG	Positive
*Laboratory values*			
Leucocytes (K/*μ*L) (4.5–13.5)	16.1	4.7	20.3
Platelets (K/*μ*L) (140–440)	252	86	243
Neutrophils (K/*μ*L) (1.30–9)	13.68	4.42	16.04
Lymphocytes (K/*μ*L) (1.90–7.5)	1.45	0.09	2.23
C-reactive protein (mg/L) (<9.9)	202.4	213	284.4
Erythrocyte sedimentation rate (mm/hour) (0–20)	46	56	50
Fibrinogen (mg/dL) (183–503)	640	501	495
D-dimers (mcg/mL) (≤0.5)	2.98	17.82	4.29
Ferritin (ng/ml) (13–145)	203	490	2574
Albumin (g/dL) (3.8–5.4)	3.0	2.5	3.4
Creatinine (mg/dL) (0.6 to 1.3)	0.56	0.62	2.08
Troponin (ng/mL) (0.00–0.030)	0.539	0.274	1.456
B-type natriuretic peptide (pg/mL) (1–100)	396	1213	383
*Chest imaging*	Not done	Chest CT scan: bilateral infiltrates with small pleural effusion	Chest radiograph: Bilateral infiltrates
*Transthoracic echocardiographic findings*	Hyperdynamic left ventricular systolic function; shortening fraction of 43.7%	Hyperdynamic left ventricular systolic function; shortening fraction of 40%	Borderline low systolic function with a shortening fraction of 29–30%. No coronary artery ectasia or aneurysms. No evidence of pulmonary hypertension. Trivial pericardial effusion. Electrocardiography (EKG): nonspecific T-wave changes and low-voltage QRS.
*Duration of ICU stay (days)*	4	12	6

## Data Availability

All data generated or analyzed during this clinical case report are included within this article.

## Abbreviations

MIS-C: Multisystem inflammatory syndrome in children
RT-PCR: Reverse transcription polymerase chain reaction
BNP: B-type natriuretic peptide.
